# Pulmonary Arterial Hypertension in Patients with Primary Sjögren's Syndrome

**DOI:** 10.1155/2014/710401

**Published:** 2014-01-09

**Authors:** Senol Kobak, Sezai Kalkan, Bahadır Kirilmaz, Mehmet Orman, Ertuğurul Ercan

**Affiliations:** ^1^Department of Rheumatology, Faculty of Medicine, Sifa University, Izmir, Turkey; ^2^Department of Internal Medicine, Çanakkale Onsekiz Mart University, Çanakkale, Turkey; ^3^Department of Cardiology, Çanakkale Onsekiz Mart University, Çanakkale, Turkey; ^4^Department of Statistic, Faculty of Medicine, Ege University, Izmir, Turkey

## Abstract

*Introduction*. Primary Sjögren's syndrome (pSS) is an autoimmune epithelitis. Pulmonary arterial hypertension (PAH) is an important and severe complication, which is encountered in many collagen tissue disorders. Early diagnostic strategies are required to define it at the asymptomatic stage. Doppler echocardiography is an important, noninvasive screening test for PAH diagnosis. *Objective*. The aim of this present study is to define the frequency of PAH in patients with pSS and to reveal correlations with laboratory and clinical findings. *Material and Methods*. A total of 47 patients, who were diagnosed with pSS according to American-European Study Group criteria were enrolled in the study. After all patients were evaluated clinically and by laboratory tests, Doppler echocardiography was performed in the cardiology outpatient clinic. Systolic pulmonary artery pressure (SPAP) >30 mm Hg values, which were measured at the resting state, were accepted as significant for PAH. *Results*. Forty-seven patients with pSS were included in the study. The mean age of patients was 48 years and the mean disease duration was 5.3 years. PAH was defined in 11 of the 47 patients (23.4%). The SPAP value was over 35 mm Hg in 5 out of 11 patients, whereas six patients had SPAP measuring 30–35 mm Hg. While pulmonary hypertension was related with earlier age and shorter duration of disease (*P* = 0.04), there was no statistically significant correlation between SPAP increase and clinical findings (*P* > 0.05). *Conclusion*. We have defined high PAH frequency in patients with pSS. Since there are different data in the literature, it is obvious that large scale, multicentre studies are required.

## 1. Introduction

Pulmonary arterial hypertension (PAH), which may progress with a fatal outcome, is characterized by increased right ventricular pressure and pulmonary vascular resistance. It may result in cardiac failure, pulmonary edema, arrhythmia, and/or sudden death [1]. It may develop secondary to an underlying disease (cardiac and lung diseases, connective tissue diseases, etc.) along with primary PAH with an unknown etiology [2]. PAH is a serious and sometimes fatal complication in various connective tissue diseases (like scleroderma, mixed connective tissue disease (MCTD), systemic lupus erythematosus (SLE), rheumatoid arthritis (RA), etc.) [3–5]. It may be encountered with an underlying interstitial fibrosis or direct proliferative vascular involvement without any parenchymal diseases [6]. Different pathogenetic mechanisms have been discussed in PAH, which is observed among connective tissue diseases. Some of these hypotheses are accumulations of immune complexes and complements on the wall of pulmonary artery, vasospasm, presence of different autoantibodies, increased serum endothelin-1 levels, and immune-mediated vascular damage [7]. Literature information about PAH prevalence encountered in connective tissue disorders is limited and contradictory. This is caused because different classification criteria and methods are used for PAH [8]. It is experienced more frequently in diffuse scleroderma (4.9%–38%), CREST syndrome (50%), RA (27.5%), and SLE (3–5%) [9–12]. There was no prospective study in the literature apart from case reports about prevalence of PAH in primary Sjögren's syndrome.

Right cardiac catheterization is the most sensitive and correct method for pulmonary artery pressure detection, but as it is an invasive procedure, it is not frequently encountered in the clinical practice. Transthoracic Doppler echocardiography (TTE) is an important noninvasive screening method for PAH detection. TTE gives information about systolic pressure of pulmonary artery by measuring systolic regurgitating tricuspid flow [13]. Sensitivity and specificity of these methods are 0.79–1.0 and 0.68–0.98, respectively. Grade of PAH prognostic variables (pericardial effusion and RV ejection fraction) and other causes (LVD and valvular cardiac disease) can be detected by TTE [14].

The aim of this present study is to investigate frequency of pulmonary hypertension in patients with primary SS by TTE method and to define any possible correlation with clinical and laboratory parameters.

## 2. Material and Methods

A total of 47 patients with pSS, who applied to the rheumatology outpatient clinic of our university hospital in one year period and were diagnosed with pSS according to American-European Study Group criteria, were included into the study. All patients were informed about the study and written consents were provided from them. After patients were evaluated clinically and by laboratory tests, Doppler echocardiography was performed in the cardiology outpatient clinic. Resting SPAP values of >30 mm Hg on TTE were accepted as significant for PAH [15]. Other causes of pulmonary hypertension (genetic, idiopathic, cardiac, and lung diseases and patients with chronic thromboembolism histories) or drugs taken (calcium channel blockers, sildenafil, etc.) were excluded, and patients with such histories were not included into the study. Also any drugs that may affect the measurements were discontinued temporarily 10 days before Doppler examination. The patients were questioned in detail with respect to exposure to environmental pollutants, such as silica and asbestos, and none of them reported exposure. SS patients with and without PAH were compared with respect to various clinical and laboratory parameters. Respiratory function tests and carbon monoxide diffusion capacity were ordered for all patients. FVC < 80% and DLCO < 70% were accepted as abnormal.

### 2.1. Laboratory Evaluation

Routine laboratory tests (hemogram, renal and hepatic function tests, and routine urine analysis) were ordered for all patients. ANA was investigated by the indirect immunofluorescent method. Anti-Ro, anti-La, and anticardiolipin antibodies were investigated by ELISA method; RF was investigated by the nephelometry method and ESR was investigated by Westergren method.

### 2.2. Chest X-Ray

A-P and lateral chest graphics with thorax HRCT were performed in all patients to exclude parenchymal lung disease, chronic obstructive pulmonary disease, pulmonary tuberculosis, and bronchogenic carcinoma.

### 2.3. Echocardiographic Evaluation

2D images and spectral and colored flow Doppler recordings were provided from all participants by using the 3.5 MHz probe of GE Vivid 7 Dimension (GE Healthcare, Norway, WI) echocardiography device. Measurements were performed while participants were lying in supine position on their left sides and accompanied by echocardiographic recordings. Measurements and evaluations of right and left ventricles were performed according to the guidelines of American echocardiography association [16]. Firstly, maximum tricuspid regurgitation (TR) was measured. TR values provided were based on single measurements only and were not normally distributed, with a variance of 102.15. The diameter of vena cava inferior (VCI) and its relationship to respiratory changes were routinely measured and, in the absence of VCI collapse, average right atrium pressure was estimated to be nearly 10 mmHg. Right ventricle pressure was calculated by adding 10 mmHg to the TR. Unless there was right ventricular outflow obstruction, this value was taken as being equal to PASP. All measured parameters were recorded at the end of expiration by blocking respiration of the participants, so that flow samples were not affected by respiration and they were more stable. Mean of sequential three recordings was calculated. Left ventricular ejection fraction was calculated after diastolic and systolic volumes were measured from the apical four-chamber view by the modified Simpson method. In evaluation of right ventricular global systolic function, tricuspid valve annular peak distance (TAPSE) measurement was calculated from apical 4-chamber cross-sections by placing in the M-mode, which was an alternative method [17]. In our cohort there was not any patients with pericardial effusion, altered interventricular septal motion or abnormal TAPSE.

### 2.4. Statistical Analysis

Cross-tables were used in the analyses of data, and according to suitable conditions in the analyses Chi-square or Fisher's exact tests were used. Data were summarized as frequency and percentages. The level of significance was accepted as *P* < 0.05.

## 3. Results

A total of 47 patients (45 of them were female) with pSS were included in the study. The mean age of patients was 48 years and the mean disease duration was 5.3 years. According to transthoracic Doppler USG findings, PAH had been detected in 11 out of 47 patients (23.4%). The SPAP value was over 35 mmHg in 5 out of 11 patients, whereas 30–35 mmHg was detected in six patients. All patients with PAH were female, the mean age was 41.6 years and the mean disease duration was 4.3 years (Table 1). When the patients were evaluated for ANA positivity, 9 out of 11 patients had granular, 1 patient had centromere, and 1 patient had homogenous type. In respiratory function tests, 2 out of 11 patients had decreased FVC and five patients had DLCO level lower than expected. When patients with pulmonary hypertension were compared with patients without pulmonary hypertension, they were younger and had shorter disease durations (mean ages and disease durations were 41.6 versus 56.2 years and 4.3 versus 9.5 years). Although no vasculitis, antiphospholipid syndrome, lung involvement and secondary lymphoma was detected in both patient groups, hypocomplementemia was detected only in 4 patients. Correlations between different parameters were examined in patients with pulmonary hypertension, and no statistically significant difference was detected (Table 2).

## 4. Discussion

Primary Sjögren's syndrome is a chronic, autoimmune disease, which can involve different organs and systems along with exocrine glands [18]. Lung involvement is one of the important extraglandular findings. Chronic bronchitis, pleuritis, interstitial pulmonary disease, and small airway diseases are some of these involvements [19]. Although it is just in the form of case reports in the literature, pulmonary artery involvement in pSS is one of the prominent findings. Some of significant clinical and laboratory findings of cases reported in pSS in the literature are mainly young females; disease duration shorter than 4 years; Raynaud's phenomenon; positivity of rheumatoid factor, anti-RNP and anti-Ro antibody; hypergammaglobulinemia and hypocomplementemia [20–22]. Sato et al. defined PAH in two patients with pSS [23]. They also demonstrated intima proliferation and plexiform lesion along with immune complex and complement accumulation in the wall of pulmonary artery in the histopathological evaluation of these two cases. Also Nakagawa et al. reported that hemodynamic and clinical improvements were observed with corticosteroid treatment in a patient with severe PAH [24]. They thought that response to corticosteroids, different proinflammatory cytokines, and vasoactive substances (endothelin-1, thromboxane A2, etc.) might be indicators for the contributions into PAH. Data related to PAH in other connective tissue diseases are contradictory [25]. While scleroderma and MCTD are more frequently encountered, pSS incidence is reported to be lower. We have defined PAH incidence in pSS as 23.4% in our study by the Doppler echocardiography method. Moreover, a possible correlation has been searched with different demographic and clinical parameters. While PAH has been related to younger age and shorter disease duration, there has been no correlation with other clinical parameters. Correlation between Raynaud's phenomenon and PAH has been reported previously in the literature [26]. While Raynaud phenomenon is reported in one out of 11 patients in our study, there was no statistical correlation. A relationship is built between presence of different autoantibodies and pulmonary hypertension. Correlation between antiphospholipid syndromes caused by anticardiolipin antibodies and PAH is well known in the literature [27]. In our study, no antiphospholipid syndrome or anticardiolipin antibody positivity has been detected. It has been believed that anti-U1RNP antibody positivity in patients with MCTD could cause more severe PAH. Again, it has been speculated that there can be a correlation between anti-Ro antibody positivity and PAH [28]. In our study, anti-Ro antibody has been detected in 7 out of 11 patients with PAH (63.6%), and it has been statistically demonstrated that there were no risk factors for PAH. In immunofluorescent studies, deposits of immunoglobulin and complement have been observed in the wall of pulmonary artery, and it has been emphasized that hypergammaglobulinemia/hypocomplementemia could be an important risk factor. While hypocomplementemia has been detected only in 2 out of 11 patients with PAH, the number of patients with detected hypergammaglobulinemia was eight.

Mechanisms of pathogenesis in pulmonary artery hypertension in pSS have not been clear yet. Some of the accused etiological factors are endothelial damage, immune complex accumulation, necrotizing vasculitis, synthesis of endothelial vasoactive molecules, and imbalances in their metabolisms [29]. Long-term vasospasm can cause an irreversible disorder as a result of structural venous changes and remodeling, stenosis of pulmonary arteries, and thrombotic obstruction. During the period, which ended up with pulmonary proliferative vasculopathy, medial hypertrophy, endothelial cellular dysfunction/proliferation, and plexiform lesions are observed. These changes are supposed to end up with progressive pulmonary artery obliteration and increased pulmonary artery pressure [30, 31].

There are some limitations in our study. Although PAH was mild and asymptomatic in the majority of the pSS patients, the frequency of 23.4% is still very high. This high frequency of PAH in pSS may be due to referral bias. More importantly, measurement of PASP by TTE may overestimate the frequency of PAH. Traditionally, TTE has been reported as a noninvasive diagnostic tool, predicting PASP similarly to cardiac catheterization. Although transthoracic Doppler echocardiography is the noninvasive and cheap method for PAH diagnosis, we actually know that right cardiac catheterization is the gold standard [32]. PAH is diagnosed depending on indirect findings by echocardiography. Therefore, pulmonary artery results, which are measured by echocardiography, can be changed in right cardiac catheterization. However, Arcasoy et al. compared the PASP estimated by TTE and measured by cardiac catheterization in 166 patients with advanced lung disease [33]. Although the correlation was good (*r* = 0.69, *P* < 0.0001), they found that 52% of the PASP measurements using TTE were inaccurate and 48% of the patients were misclassified as having PAH if the diagnosis was based on TTE alone. Also, PAH treatment option can be defined by the vasoreactivity test performed during right cardiac catheterization. It might have been possible to define PAH frequency by comparing these two methods in our study. However, Doppler echocardiography should be considered as a screening method in connective tissue diseases. Pulmonary hypertension defined in the echocardiography should be confirmed by performing the right cardiac catheterization.

In conclusion, we have detected high PAH frequency in asymptomatic pSS by using Doppler echocardiography. The message is that, using TTE, mild elevations in PASP are not uncommon in pSS. Although estimation of PASP by TTE is not as reliable as cardiac catheterization, it is possible that mild PAH may contribute to the high incidence of cardiovascular related deaths in pSS. Further multicentre investigations will be required regarding the issues of early diagnostic possibility and new treatment strategies.

## Figures and Tables

**Table 1:** Clinical and laboratory findings of 11 patients with pulmonary artery hypertension.

Patients no.	Age/ gender	Dry eyes	Dry mouth	CNS	Raynaud	SPAP mmHg	DLCO %	FVC %	ANA	Anti-Ro	Anti-La
1	42/f	+	+	−	−	42	67	78	Gran.	+	−
2	43/f	−	+	−	−	34	70	86	Gran.	+	−
3	45/f	+	+	−	+	32	88	92	Gran.	+	−
4	51/f	+	+	−	−	34	90	96	Homog.	+	+
5	38/f	+	+	+	−	35	80	92	Gran.	−	−
6	41/f	+	+	−	−	31	96	98	Gran.	+	+
7	39/f	+	+	−	−	40	65	76	Sentr.	−	+
8	42/f	+	−	−	−	38	68	70	Gran.	+	−
9	37/f	+	+	−	−	39	68	72	Gran.	−	−
10	40/f	+	+	−	−	31	92	90	Gran.	+	−
11	40/f	+	−	−	−	34	86	94	Gran.	−	+

**Table 2:** Comparison of demographic and clinical features in pSS patients with and without pulmonary artery hypertension.

	pSS with PAH (*N* = 11, 23.4%)	pSS without PAH (*N* = 36, 76.6%)	*P* value
Age (mean)	41.6	59.2	0.04
Duration of disease (years)	4.3	9.5	0.04
Dry eyes	10 (90.9%)	29 (80.6%)	0.424
Dry mouth	9 (23.1%)	30 (76.9%)	0.907
Arthralgia	9 (81.8%)	33 (91.7%)	0.354
CNS involvement	1 (9.1%)	0 (0%)	0.675
Raynaud's phenomenon	1 (9.1%)	7 (19.4%)	0.424
Anti-Ro Ab	7 (63.6%)	21 (58.3%)	0.754
Anti-La Ab	4 (36.4%)	16 (44.4%)	0.635
C-reactive protein	3 (27.3%)	8 (22.2%)	0.729
ESR	5 (45.5%)	13 (36.1%)	0.577
